# Marker-Assisted Pyramiding of Downy Mildew-Resistant Gene *Ppa3* and Black Rot-Resistant Gene *Xca1bo* in Popular Early Cauliflower Variety Pusa Meghna

**DOI:** 10.3389/fpls.2021.603600

**Published:** 2021-08-23

**Authors:** Partha Saha, Chandrika Ghoshal, Namita Das Saha, Aakriti Verma, Mohita Srivastava, Pritam Kalia, Bhoopal Singh Tomar

**Affiliations:** ^1^Division of Vegetable Science, Indian Council of Agricultural Research-Indian Agricultural Research Institute, New Delhi, India; ^2^Centre for Environment Science and Climate Resilient Agriculture, Indian Council of Agricultural Research-Indian Agricultural Research Institute, New Delhi, India; ^3^Division of Genetics, Indian Council of Agricultural Research-Indian Agricultural Research Institute, New Delhi, India

**Keywords:** cauliflower, black rot, downy mildew, MAS, resistance

## Abstract

Cauliflower is an important extensively grown cool season vegetable in India. Black rot and downy mildew are major devastating diseases reducing yield and quality of the crop. To tackle these through host plant resistance, a marker-assisted backcross breeding method was followed to pyramid a black rot-resistant gene (*Xca1bo*) and a downy mildew-resistant gene (*Ppa3*) from donors BR-161 and BR-2, respectively, into the background of Pusa Meghna cauliflower cultivar. Marker-assisted backcross breeding was followed up to BC_2_ generation using SCAR marker ScOPO-04_833_ and SSR marker BoGMS0624 for black rot and downy mildew resistance genes in foreground selection, respectively. In background selection, at each stage of backcrossing, 47 parental polymorphic SSR markers were used. The graphical genotyping of the five two-gene (*Xca1boXca1boPpa3Ppa3*) homozygous BC_2_F_2_ plants showed an average recovery of 85.44% of the Pusa Meghna genome with highest genome recovery of 91.7%. The genome contribution of donor parents (BR-161 and BR-2) was 8.26 with 6.34% of residual heterozygousity. The backcross derived pyramided lines BC_2_F_2:3-7-16_ and BC_2_F_2:3-7-33_ showed high resistance to both the diseases and exhibited higher yield and vitamin C content as compared with recipient parent Pusa Meghna. It is, therefore, evident from this study that resistant genes can be introgressed successfully into a Pusa Meghna cultivar without any yield penalty, benefitting farmers with reduced input cost and consumers with chemical residue free produce. Besides, the pyramided lines carrying dominant resistant genes can be exploited in a hybridization programme to develop hybrid(s) in cauliflower.

## Introduction

Cauliflower (*Brassica oleracea* var. *botrytis* L.) is one of the most popular vegetables in the Brassicaceae family because of its high nutritive value as well as value added foods in the processing sector (Varalakshmi et al., 2011; Vanlalneihi et al., 2020). This herbaceous annual plant develops “curd” composed of highly suppressed pre-floral apical meristem, which is the main edible part (Sidki, 1962). Cauliflower originated in the island of Cyprus from where it was taken to Middle East and North Western Europe (Boswell, 1949). Dr. Jemson, a famous botanist at Kew garden, London, United Kingdom, the then In-charge of the Company Bagh (United Provinces, Saharanpur in the Northern plains of India), introduced cauliflower in India in the year 1822. Since then, cauliflower has been grown by farmers in North Indian plains from May to July (hot and humid weather). Farmers raised their own seeds from selected survived plants for raising next season crop and continued this process year after year, which led to adaptation of these new types to hot and humid climate later known as having wavy leaves, loosely arranged broad with short or long stalk Indian cauliflower (Swarup and Chatterjee, 1972). This plant matures from mid-September to mid-October and is sold at high market prices.

China is the leading country for production of cauliflower followed by India, Spain, Italy, France, United States, and United Kingdom. India has very low productivity (19.76 MT/ha) in contrast to other countries (Egypt, 29.93 MT/ha; Italy, 24.73 MT/ha; Poland 22.29 MT/ha) (FAO, 2014). The low productivity is mainly due to susceptibility of commercial varieties and hybrids to various biotic and abiotic stresses. Black rot and downy mildew are the two most devastating diseases that cause tremendous quality and yield loss in cauliflower, and these are predominant in most cauliflower-growing states of India (Singh et al., 2012, 2015; Saha et al., 2014, 2016, 2020; Verma and Singh, 2018). Black rot disease causing bacteria *Xanthomonas campestris* pv. *campestris* enter inside the edge of the leaves through hydathodes or wounds. The disease proliferates through vascular tissue by clogging the vessels and produces V shaped chlorotic lesions. The color of the infected veins turns black with chlorotic or necrotic interveinal tissue. Affected plants develop small-sized curds resulting in huge crop loss to farmers (Vicente et al., 2001; Doullah et al., 2011; Vicente and Holub, 2013; Saha et al., 2014; Kalia et al., 2017; Bernabé et al., 2019; Sheng et al., 2020). Downy mildew caused by obligate fungal parasite *Hyaloperonospora parasitica* (Pers. Fr.) Fr. (Giovannelli et al., 2002) is another important disease of cauliflower, which damages seedlings as well as mature crops (Laemmlen and Mayberry, 1984; Verma and Thakur, 1989; Singh et al., 2012; Verma and Singh, 2018; Saha et al., 2020). This also damages the crop in the postharvest stage (Lund and Wyatt, 1978; McKay et al., 1992). The disease reduces yield as well as curd quality because of adult plant defoliation; and if the damage is severe, the produce becomes unmarketable. Butler (1918) first reported this disease in India and presently, it is predominant in most cauliflower growing-states of India. Both the diseases, i.e., black rot and downy mildew, can be managed through cultural practices and using bactericide or fungicide, respectively (Thakur and Mathur, 2002). Nevertheless, these practices are time-consuming besides chemicals being environment polluter and health hazardous. Therefore, most sustainable approach is exploration of host plant resistance to develop resistant varieties that can offer a reliable and practical solution for effective disease management. The development of high-yielding cauliflower with multiple disease resistance through conventional backcross methods requires 8–9 years or more. Marker-assisted selection (MAS) is a simple method for transferring gene(s)/QTL(s) effectively and precisely besides hastening the process of varietal development. Resistant gene-linked molecular markers can provide another powerful approach to pyramid various resistant gene(s) into a single variety (Das and Rao, 2015; Arunakumari et al., 2016; Nguyen et al., 2018; Hsu et al., 2020).

Black rot resistance is governed by a single dominant gene at the seedling stage in the SN 445 genotype and at the adult plant stage in the BR-161 and BR-207 genotypes of cauliflower (Jamwal and Sharma, 1986; Saha et al., 2014, 2016). There are many R genes/QTLs as well as co-segregating DNA markers for resistance to black rot disease reported in cauliflower and other related crops (Camargo et al., 1995; Soengas et al., 2007; Kaur et al., 2009; Doullah et al., 2011; Kifuji et al., 2013; Tonu et al., 2013; Saha et al., 2014; Lee J. et al., 2015; Lee Y. G. et al., 2015; Sharma et al., 2016; Bernabé et al., 2019). SCAR markers linked to the black rot-resistant gene *Xca1bo* in cauliflower were developed by Kalia et al. (2017). Downy mildew resistance is governed by a single dominant gene in cauliflower (Mahajan et al., 1995; Jensen et al., 1999; Farnham et al., 2001; Verma and Singh, 2018; Saha et al., 2020) and broccoli (Coelho and Monteiro, 2003); two independent dominant genes (Carvalho and Monteiro, 1996), and three to four complementary dominant genes (Hoser Krauze et al., 1995). Previous workers also tagged downy mildew-resistant genes (Agnola et al., 2000; Farinhó et al., 2000, 2007). A resistance locus at the cotyledon stage was reported by Giovannelli et al. (2002) that led to the identification of some putative-resistant gene homologs (Gao et al., 2003). A monogenic dominant locus *Pp523* was identified in adult plants of broccoli by Coelho and Monteiro (2003). Four closely linked molecular markers along with a marker linked in repulsion phase were also developed (Farinhó et al., 2004). Singh et al. (2013) identified three markers linked to a downy mildew resistant-gene (*Ppa3*). Very recently, SSR markers linked to the downy mildew resistant-gene were identified (Saha et al., 2020).

Pusa Meghna, an early maturity group Indian cauliflower is susceptible to both black rot and downy mildew diseases causing huge losses to the farmers. Therefore, this study was under taken with the aim to pyramid genes resistant to black rot (*Xca1bo*) and downy mildew (*Ppa3*) diseases in Pusa Meghna through marker-assisted backcross breeding. A large number of mapped cauliflower or other *Brassica* specific microsatellite markers (>1,500) were available at the time when this program was initiated (Lowe et al., 2004; Li et al., 2011). These are sequence-specific co-dominant markers ideal for background selection. These allow accurate determination of the allelic arrangement of the offspring by detecting alleles coming from both the parents. Through marker-assisted breeding, it is expected that both black rot- and downy mildew-resistant loci will be introgressed into Pusa Meghna Indian cauliflower, and that the inherent morphological traits will be maintained.

## Materials and Methods

### Plant Materials and Development of Black Rot and Downy Mildew Resistant Lines

**Figure 1:**
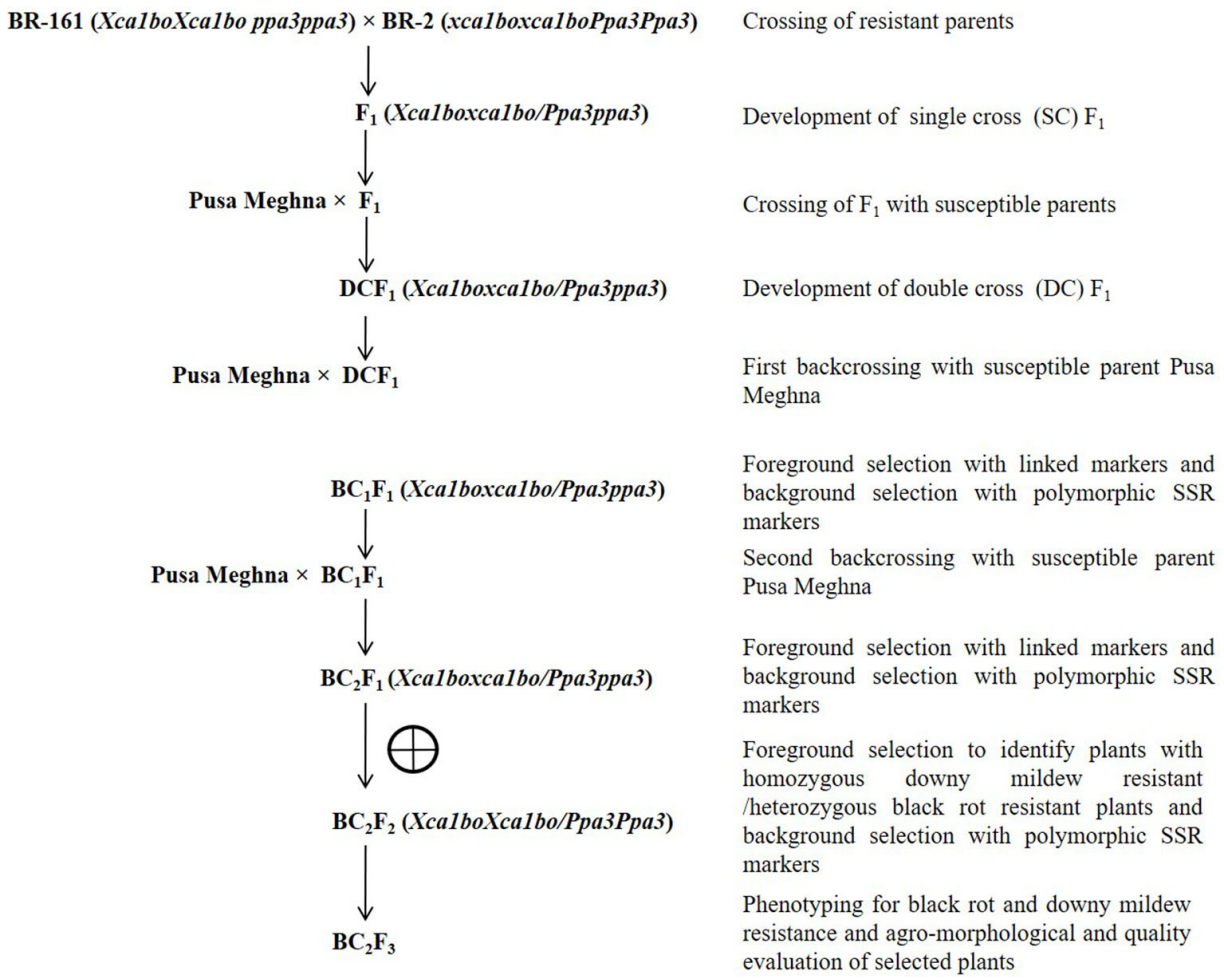
Marker-assisted backcross breeding (MABB) strategy.

Pusa Meghna, an early maturity group cauliflower variety with excellent curd quality, was used as the recurrent parent. It forms white compact medium sized curd (350–400 g) with an average yield of 175 q/ha. The genotypes BR-161 (carrying black rot-resistant gene *Xca1bo*) and BR-2 (carrying downy mildew-resistant gene *Ppa3*) were used as donor parent for black rot- and downy mildew disease-resistant genes, respectively (Singh et al., 2013; Saha et al., 2014). The genotypes BR-161 and BR-2 are the homozygous inbred lines maintained at the Division of Vegetable Science, ICAR-Indian Agricultural Research Institute, New Delhi, India. For targeted introgression of *Xca1bo* and *Ppa3* genes into Pusa Meghna, marker-assisted a backcross breeding (MABB) strategy was followed (Figure 1). Initially, both the resistant donor parents were crossed among themselves and the resultant F_1_ (BR-161 × BR-2) was crossed with Pusa Meghna. The resultant double cross F_1_ {Pusa Meghna × (BR-161 × BR-2)} was backcrossed with recurrent parent “Pusa Meghna” and a BC_1_F_1_ population was generated. Foreground selection was performed for the desired resistant genes (*Xca1bo* and *Ppa3*), and the background analysis was conducted to know the recurrent parent genome recovery. The selected plants having maximum recurrent parent genome recovery along with the desired resistant genes were again backcrossed with Pusa Meghna and BC_2_F_1_ generation was generated. The best plant was selected and selfed to develop BC_2_F_2_ generation. To identify homozygous black rot-resistant plant in the BC_2_F_2_ generation, BC_2_F_3_ plants were generated by selfing each BC_2_F_2_ plant. In every backcross generation, the plants that showed the heterozygous allele for BR-161 and BR-2 were selected. In the BC_2_F_3_ population, five homozygous plants carrying both resistant genes along with a maximum genome of Pusa Meghna were selected finally.

### DNA Isolation and Marker Assisted Foreground Selection for Black Rot- and Downy Mildew-Resistant Genes

CTAB method with slight modifications was used for isolating genomic DNA of parents and that of each backcross generation plants (Doyle and Doyle, 1990). The closely linked SCAR marker ScOPO-04_833_ linked to the *Xca1bo* gene and SSR marker BoGMS0624 linked to the *Ppa3* gene were used for selecting the target genes under foreground selection. The SCAR marker ScOPO-04_833_ was previously developed by Kalia et al. (2017) and is linked to the black rot-resistant locus *Xca1bo* at 1.6 cM distance. The SSR marker BoGMS0624 was previously identified from the 92 RIL population of Pusa Himjyoti × BR-2 (unpublished data). This marker is linked to the downy mildew-resistant locus *Ppa3* at 3.26 cM distance. Backcross plants (BC_1_F_1_) were screened with the SCAR marker ScOPO-04_833_ to identify plants carrying the *Xca1bo* gene for black rot disease resistance. The sequences of primers are mentioned in Table 1. PCR and gel electrophoresis protocols followed by Williams et al. (1990) and Kalia et al. (2017) were used for the marker-assisted selection of *Xca1bo* and *Ppa3*, respectively. The plants that had the marker for black rot resistance in the BC_1_F_1_ population were then screened with a BoGMS0624 marker for the presence of the *Ppa3* gene under heterozygous conditions.

**Table 1:** SCAR and SSR markers used for foreground selection of the black rot (*Xca1bo*) and downy mildew resistance (*Ppa3*) genes.

**Genes**	**Markers**	**Linkage Groups (LG)**	**Forward sequences**	**Reverse sequences**	**Annealing Temperature (AT) (^**°**^C)**	**Sources**
*Xca1bo*	ScOPO-04_833_	3	ACGTAGCGTCCTGGAGCGAAGTG	ACGTAGCGTCAGCAAAGATCATGGCTC	58	Kalia et al., 2017
*Ppa3*	BoGMS0624	9	AAGACGAAGTCAAGTCAAGGT	CGTATCATCCAGAGTATCCAG	55	Unpublished

### Parental Polymorphism Survey for Background Analysis of Pyramided Lines

The polymorphism survey was carried out using previously reported 225 SSR markers (Li et al., 2011). The chromosome-wise markers used for polymorphism survey are given in Table 2. The PCR protocol was followed as per Saha et al. (2020). The identified BC_1_F_1_ plants were genotyped with 47 parental polymorphic SSR markers. The same steps as described above were followed in BC_2_F_1_ generation. In the BC_2_F_2_, generation, the plants were screened with the linked markers to identify resistant plants showing homozygous condition. These plants were then screened using parental polymorphic SSR markers for the identification of the best BC_2_F_2_ plants. As the SCAR marker for the black rot-resistant gene *Xca1bo* is dominant in nature, progeny testing/phenotyping in BC_2_F_3_ was performed to identify plants carrying *Xca1bo* under homozygous conditions in BC_2_F_2_.

**Table 2:** Chromosome-wise microsatellite markers used for polymorphism survey between the parents Pusa Meghna, BR-161 and BR-2, and the number of polymorphic markers.

**Chromosomes**	**Total no. of markers analyzed**	**No. of polymorphic markers between Pusa Meghna and BR-161**	**No. of polymorphic markers between Pusa Meghna and BR-2**
1	13	3	5
2	31	3	8
3	48	4	6
4	14	4	5
5	8	0	0
6	16	1	2
7	29	7	6
8	31	2	5
9	35	1	1
Total no. of markers	225	25	38

### Raising of Parents and Pyramided Lines

In the research farm of Division of Vegetable Science, ICAR-Indian Agricultural Research Institute, New Delhi, India, all parental lines, F_1_, BC_1_F_1_, BC_2_F_1_, BC_2_F_2_, and BC_2_F_3_ were grown. The research farm is situated at an altitude of about 228.61 m above mean sea level, with 28°08′N latitude and 77°12′E longitude. The climate is sub-temperate and semiarid, and the soil type is alluvial. Summer is very hot and relatively dry, with an average temperature of above 40°C, and winter is cool with a temperature of below 5°C.

During the 1 week of July, the seeds were sown in nursery and 1-month-old seedlings were transplanted in the main field with 60 cm distance between the rows and 45 cm within rows. Recommended package of practices were followed to raise the crop as described by Thamburaj and Singh (2003). Before transplanting, 22 tons of farmyard manure; 55 kg N: 70 kg P: 60 kg K per hectare were applied to the field. A month after transplanting, a dose of 35 kg N was applied again at the time of first earthing up. During curd initiation, a similar dose of N was applied at second earthing up. Before transplanting, Stomp (Pendimethalin @ 3.5 l/hectare) was applied for controlling the weed. For maintaining sufficient humidity, broad bed furrow method of irrigation was applied at a fortnight interval.

### Screening for Black Rot Resistance in BC_2_F_3_

The strain of *Xanthomonas campestris* pv. *campestris* race 1 (isolate number *Xcc*-C 1) was used for screening the parents and BC_2_F_3_ population. The isolate was provided by Dr. Dinesh Singh, Division of Plant Pathology, ICAR-Indian Agricultural Research Institute, New Delhi, India. The bacterial culture having 0.1 optical density at 600 nm was diluted to 19 × 10^8^ CFU ml^−1^ before inoculation. The first inoculation was done at 15 days after transplanting and the second at 45 days after transplanting. Three inner whorl young leaves were selected in each plant and inoculated by following leaf cut and dip technique as described by Kapoor et al. (1985). The scoring of inoculated plants was done in a scale of 0–4 for three times in each inoculation at 20 days interval as per Tewari et al. (1987). The results were observed after 60 days of inoculation, and infected leaves were scored accordingly. The disease score was 0 for no visible symptom, 1 for minute symptom, (one or two tiny lesions at the leaf edge), and 2 was for a handful medium-sized slow-progressing “V” shaped lesions spreading up to 20% of leaf area, which were rated as resistant. On the other hand, 3 was scored for numerous large V-shaped lesions spreading almost half of the leaves, which resulted in leaf wilting and distortion, and 4 for major infection spreading more than 75% of the entire leaf area, which leads to the wilting of leaves, scored as susceptible.

### Screening for Downy Mildew Resistance in BC_2_F_3_

The conidial suspension of *H. parasitica* Delhi isolate at 5 × 10^3^ spores ml^−1^ was used for artificial inoculation of parents and BC_2_F_3_ plants in the field (Mahajan et al., 1995). The inoculum was sprayed on both sides of all leaves using a pneumatic knapsack sprayer at 30 days after transplanting. All diseased leaves of the downy mildew-susceptible parent were retained to increase the disease pressure in the infector rows of the experimental block. A five-point scale, as suggested by Mahajan et al. (1995) and modified by Pandey et al. (2001), was used to score the plants 15 days after inoculation. The percentage disease incidence (DI) was calculated by observing the infected areas of each leaf along with infected leaves per plant. The plants were categorized as resistant (R; 1 < DI ≤ 10); moderately resistant (MR; 10 < DI ≤ 25); moderately susceptible (MS; 25 < DI ≤ 50); susceptible (MS; 50 < DI ≤ 75), or highly susceptible (HS; 75 < DI ≤ 100) on the basis of their DI values.

### Microstructure Analysis of Pyramided Lines

For microstructure analysis, the STRUCTURE 2.3.4 software, which uses a systematic Bayesian clustering approach based on Markov chain Monte Carlo (MCMC) estimation, was utilized to study the fundamental genetic structure within pyramided lines (Pritchard et al., 2000). K, the number of presumed populations, was set from 1 to 10. For each run, the burn-in period was set to 10,000, and MCMC (Markov Chain Monte Carlo) was set to 1; 50,000 each with iterations going up 7. The value of K in DK (log probability for the rate of change of the data) by the Evanno Test (Evanno et al., 2005) was identified to determine the optimal number of subgroups in the lines. To visualize the outputs, “Structure Harvester” was used from which plots, such as Evanno plots, were constructed.

### Evaluation for Agro-Morphological and Quality Traits of the Pyramided Lines

The selected BC_2_F_3_ plants along with the parental lines were evaluated for 2 years in replicated trials. Twenty plants in each of pyramided lines were planted in a randomized block design (RBD) at a spacing of 60 × 45 cm at the research farm of Division of Vegetable Science, ICAR-Indian Agricultural Research Institute, New Delhi, India. The details of experimental location and package of practices are already given in the section of raising of parents and pyramided lines. Observations were recorded from three randomly selected plants in each line for five agro-morphological and yield related traits viz., plant height (cm), days to 50% curd maturity, curd angle (°), marketable curd weight (kg), curd yield (q/ha) as per distinctness, uniformity, and stability (DUS) guidelines of cauliflower (PPV and FRA, 2009). Vitamin C was estimated from the curd at the edible stage of maturity as per the protocol with some modifications as given by Jagota and Dani (1982) and Vanlalneihi et al. (2020).

### Statistical Analysis

The segregation of markers in the BC_2_F_2_ population was analyzed for goodness-of-fit by chi square (χ2) analysis (Panse and Sukhatme, 1967). To determine the significant variation among the pyramided lines for yield and quality traits, ANOVA was performed online (http://14.139.232.166/opstat/onefactor.htm). To calculate the amount of recurrent parent (Pusa Meghna) genome contribution in pyramided lines, software programme Graphical genotypes (GGTs) version 2.0 was used (van Berloo, 2008).

## Results

### Parental Polymorphism Survey for Foreground and Background Selection

During parental polymorphism survey using linked markers, the SCAR marker ScOPO-04_833_ amplified fragments of ~833 bp in the resistant parent (BR-161), but was absent in susceptible parent Pusa Meghna. With respect to the SSR marker BoGMS0624, a band of 350 bp fragment was amplified in BR-2 compared with that of Pusa Meghna in which a fragment of 320 bp was amplified. Thus, resistant lines were distinguished from susceptible one using linked markers. Out of 225 SSR markers used for parental polymorphism survey for background selection, 25 markers indicated polymorphism (11.11%) between Pusa Merghna and BR-161, and 38 (16.88%) markers were polymorphic between Pusa Meghna and BR-2 (Table 2).

### Introgression of Black Rot- and Downy Mildew-Resistant Genes Into Pusa Meghna Background

**Figure 2:**
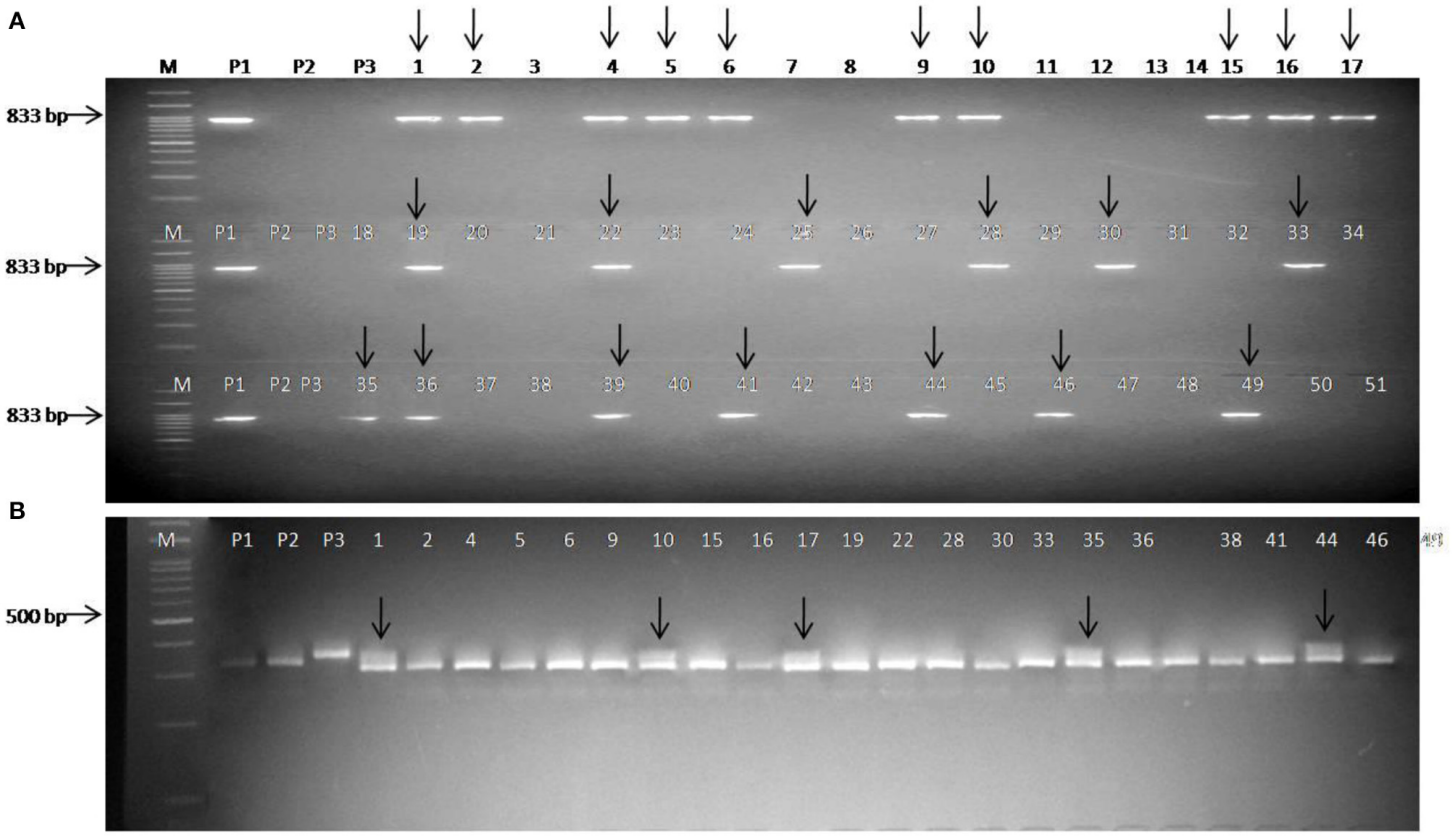
Foreground selection in BC_1_F_1_ plants. **(A)** Screening of plants with the SCAR marker ScOPO-04_833_ and arrow indicates the plants positive for black rot-resistant gene *Xca1bo*; **(B)** screening of *Xca1bo*-positive plants using *Ppa3*-linked marker BoGMS0624 and arrow indicates positive for downy mildew-resistant gene *Ppa3*; Lanes M: 100 bp ladder; P1: donor (BR-161) for black rot-resistant gene; P2: Pusa Meghna (recurrent parent); P3: donor (BR-2) for downy mildew-resistant gene.

A total of 51 BC_1_F_1_ plants were first checked for the presence of the black rot-resistant gene *Xca1bo* using linked SCAR marker ScOPO-04_833_. Out of these, 23 plants were found positive for black rot resistance (*Xca1boxca1bo*), and these plants were checked for the presence of the downy mildew-resistant gene (*Ppa3*) under heterozygous (*Ppa3ppa3*) conditions using linked SSR marker BoGMS0624. Finally, five BC_1_F_1_ plants were found to be resistant for both black rot and downy mildew diseases (Figure 2). These five plants were compared with the recurrent parent Pusa Meghna in field for morphological traits and, finally, selected for background analysis, and the plants having maximum recurrent parent genome contribution were identified. A total of 65 BC_2_F_1_ plants were tested for the presence of black rot-resistant gene, out of which 30 plants were found positive for SCAR marker ScOPO-04833. These lines were then tested for the presence of downy mildew-resistant gene and, finally, 14 plants were found heterozygous for the downy mildew-resistant gene. Out of the 14 plants, five were selected based on their maximum phenotypic similarity with recurrent parent Pusa Meghna.

A total of 102 BC_2_F_2_ plants were screened using the black rot resistant-gene (*Xca1bo*)-linked SCAR marker ScOPO-04833 to identify plants that are positive for black rot resistance, and, finally, 78 plants were found to show resistant parent specific band. The marker segregated into 3:1 ratio (78 homozygous/heterozygous resistant: 24 homozygous susceptible) with a chi square (λ^2^) value of.11 (*P* = 0.7–0.8). Then, the 78 black rot-resistant plants were checked for the downy mildew-resistant gene (*Ppa3*) using linked SSR marker BoGMS0624. Out of these 78 plants, 21 homozygous-resistant plants, 42 heterozygous-resistant plants, and 15 homozygous-susceptible plants were observed, which segregated in the ratio of 1:2:1 (HoR:HeR:HoS) with (λ^2^) value of 1.38 (*P* = 0.5–0.7). Both markers exhibited similarity to the expected marker segregation ratio (3:1/1:2:1) (Table 3).

**Table 3:** Segregation of SCAR (ScOPO-04_833_) and SSR (BoGMS0624) markers in BC_2_F_2_ plants.

**Markers**	**Total plants screened**	**Homozygous resistant (HoR) plants**	**Heterozygous resistant (HeR) plants**	**Homozygous susceptible (HoS) plants**	**Chi square (**χ^2^**) value**	**Probability**
ScOPO-04_833_	102	78 (HoR/HeR)		24	0.11	0.70–0.80
BoGMS0624	78	21	42	15	1.38	0.50–0.70

### Phenotypic Evaluation of BC_2_F_2:3_ Plants for Black Rot Resistance

In order to identify the plants carrying the black rot resistant-gene under homozygous conditions, a phenotypic screening of BC_2_F_2:3_ progenies of 21 BC_2_F_2_ plants (carrying the black-rot resistant gene in homozygous/heterozygous form and the downy mildew-resistant gene in homozygous form) was carried out. Out of 21 progeny rows, 16 segregated into resistant and susceptible plants, and five progeny rows were resistant, which were finally selected as homozygous for the black rot-resistant gene (*Xca1boXca1bo*). The data of black rot resistance of the five homozygous plants showed resistant reaction (Table 4). The susceptible parent Pusa Meghna showed a PDI value of 4, whereas the resistant parent BR-161 showed 0 PDI value. As compared with Pusa Meghna, the pyramided lines showed a PDI value of 0–1.63. During phenotyping, there were very few symptoms or no symptom of the black rot disease on pyramided lines. The lines BC_2_F_2:3-7-16_ and BC_2F2:3-7-52_ showed a PDI value of 0, and BC_2_F_2:3-7-33_, BC_2_F_2:3-7-9_, and BC_2_F_2:3-7-72_ showed a PDI value of 0.28, 0.5, and 1.63, respectively. They also exhibited morphological characteristics similar to those of susceptible parental line Pusa Meghna.

**Table 4:** Screening of two-gene positive BC_2_F_2:3_ plants for resistance against black rot and downy mildew disease.

**Parents/Pyramided lines**	**Allelic status of *Xca1bo* and *Pap3***	**PDI for black rot**	**DI for downy mildew**
Pusa Meghna	*xaca1boxca1bo/ppa3ppa3*	4	98
BR-161	*Xca1boXca1bo/ppa3ppa3*	0	–
BR-2	*xca1boxca1bo/Ppa3Ppa3*	–	0
BC_2_F_2:3-7-9_	*Xca1boXca1bo/Ppa3Ppa3*	0.5	0
BC_2_F_2:3-7-16_	*Xca1boXca1bo/Ppa3Ppa3*	0	10
BC_2_F_2:3-7-33_	*Xca1boXca1bo/Ppa3Ppa3*	0.28	0
BC_2_F_2:3-7-52_	*Xca1boXca1bo/Ppa3Ppa3*	0	20
BC_2_F_2:3-7-72_	*Xca1boXca1bo/Ppa3Ppa3*	1.63	0

*PDI, plant disease index; DI, disease incidence*.

### Phenotypic Evaluation of BC_2_F_2:3_ Plants for Downy Mildew Resistance

The phenotyping of 5 BC_2_F_2:3_ lines, which were homozygous for downy mildew resistance, were screened phenotypically for downy mildew resistance along with parents as per the protocol described in Materials and methods. The parent Pusa Meghna was highly susceptible to the downy mildew disease with a DI value of 98%, whereas resistant parent BR-2 was free from the disease with a DI value of 0. Among the pyramided lines, all were found to be resistant and DI ranged from 0 to 10 (Table 4) and three lines, viz. BC_2_F_2:3-7-9_, BC_2_F_2:3-7-33_, and BC_2_F_2:3-7-72_ had, 0 DI; whereas two lines, namely BC_2_F_2:3-7-16_ and BC_2_F_2:3-7-52_, had a DI value of 10 and 20, respectively.

### Background Genome Analysis of Backcross Derived Black Rot and Downy Mildew Resistant Lines

**Figure 3:**
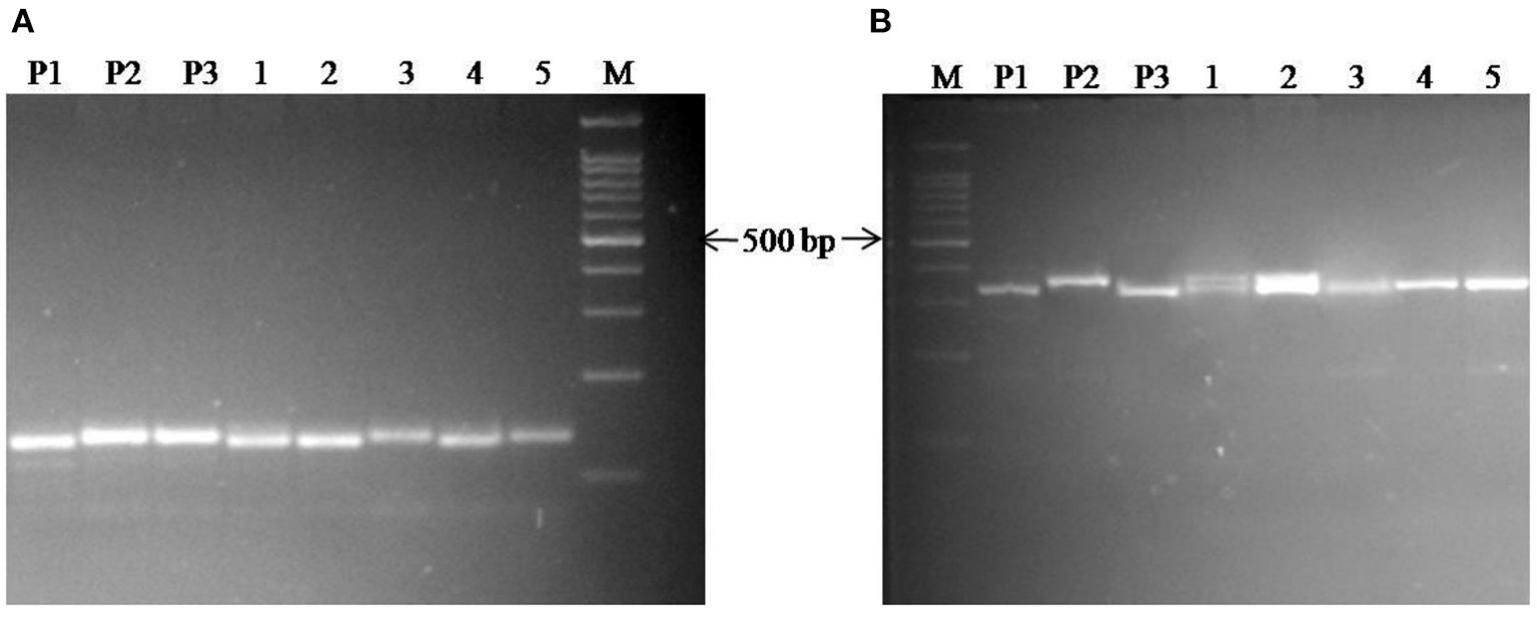
Background analysis of 5 BC_2_F_2_ plant with the polymorphic markers **(A)** for SSR marker BoGMS0836; **(B)** for SSR marker BoGMS1020; Lanes M: 100 bp ladder; P1: Pusa Meghna (recurrent parent); P2: donor (BR-161) for black rot-resistant gene; P3: donor (BR-2) for downy mildew-resistant gene; Lanes 1–5 are the BC_2_F_2_ plants.

**Figure 4:**
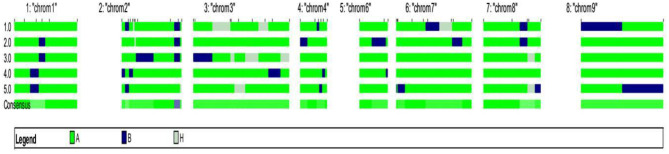
Chromosome-wise recurrent genome recovery of the five selected plants in BC_2_F_2_ generation.

**Figure 5:**
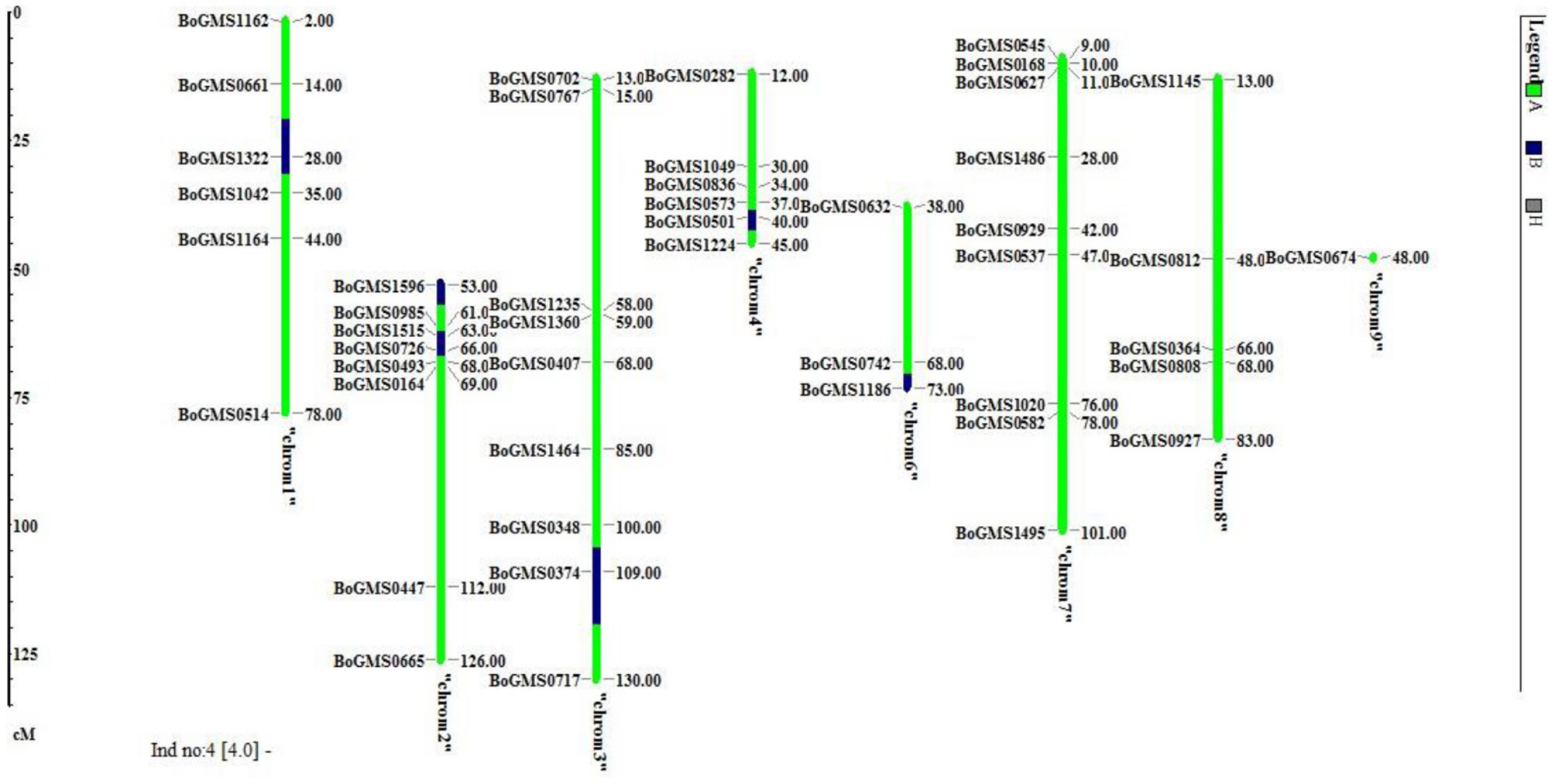
Chromosome-wise recurrent genome recovery of the individual plant number 4 in BC_2_F_2_ generation.

The background analysis of the five BC_1_F_1_ plants was carried out using 47 parental polymorphic SSR markers. The number of double gene heterozygotes/homozygote plants in the BC_1_F_1_, BC_2_F_1_, and BC_2_F_2_ populations and the maximum recurrent parent genome contribution to the selected plants are provided in Table 5. It was observed that in BC_1_F_1_ generation, the genome recovery ranged from 60.56 to 69.7%, and plant number 4 had the maximum recovery. The selected plants were backcrossed for BC_2_F_1_ generation. The background analysis of the 5 BC_2_F_1_ showed that the extent of genome recovery ranged from 81.78 to 86.5% with maximum genome recovery in plant number 3 (86.5%) followed by plant number 5 (85.69%). The background analysis of five BC_2_F_2_ plants with polymorphic SSR markers BoGMS0836 and BoGMS1020 is shown in Figure 3. For SSR marker BoGMS0836, plant numbers 1, 2, and 4 showed Pusa Meghna-specific band. In the case of SSR marker BoGMS1020, individuals 3, 4, and 5 had Pusa Meghna-specific band. The graphical genotyping of the five two-gene homozygous BC_2_F_2_ plants using Graphical genotypes (GGTs) version 2.0 is shown in Figures 4, 5. The analysis revealed an average recovery of 85.44% of the Pusa Meghna genome, whereas the recovery of BR-161 and BR-2 genomes was 8.26 with 6.34% residual heterozygosity. Background analysis on the basis of linkage groups revealed that on all the chromosomes, at least some individuals showed short segments from donor parents ranging from 1.1 to 19.4% inheritance (Figure 4, Table 6). Highest genome recovery of 97% from Pusa Meghna was observed on chromosome 6. The genome recovery of five selected lines ranged from 80.9 to 91.7%. Highest genome recovery of 91.7% of Pusa Meghna was observed in individual 4, followed by 86.4 and 84.9% of recovery in individuals 5 and 3, respectively. The chromosome-wise recurrent parent genome recovery of plant number 4 is given in Figure 5. Individual 4 inherited chromosomes 7, 8, and 9 entirely from Pusa Meghna, whereas short segments were derived from donor parents on chromosomes 1, 2, 3, 4, and 6.

**Table 5:** Number of double resistance gene heterozygotes/homozygous plants identified and estimation of recurrent parent genome recovery.

**Generations**	**Number of plants screened for**		**Number of double gene heterozygous plants**	**No. of double gene homozygous plants**	**Estimated maximum background recovery of recurrent parent (%)**	**Expected background recovery of recurrent parent (%)**
	***Xca1bo***	***Ppa3***				
BC_1_F_1_	51	23	5	–	69.7	75
BC_2_F_1_	65	30	14		86.5	87.5
BC_2_F_2_	102	78	–	5	91.7	–

**Table 6:** The analysis of introgressed and background recovery of BC_2_F_2_ plants.

**Individual plants**	**A (%)**	**B (%)**	**H (%)**	**Total (cM)**	**H-segments**
1	83.3	6.5	10.3	496	4
2	80.9	11.5	7.6	496	2
3	84.9	7.1	8.1	496	4
4	91.7	8.3	0	496	0
5	86.4	7.9	5.7	496	3
Average	85.44	8.26	6.34	496	2.6

*A, recurrent; B, donor; H, heterozygous; cM, centimorgan*.

### Microstructure Analysis of Pyramided Lines

**Figure 6:**
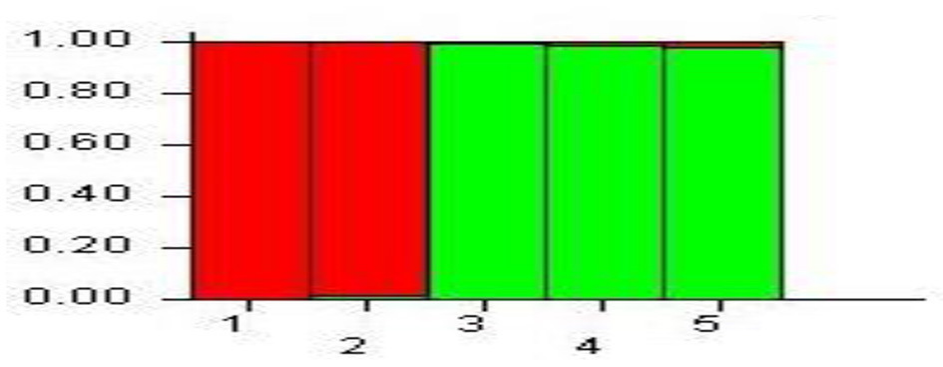
Microstructure analysis of five individual plants of BC_2_F_2_ generation (1: individual plant number 1; 2: individual plant number 2; 3: individual plant number 3; 4: individual plant number 4; 5: individual plant number 5.

Evanno test denoted the optimum *K*-value by observing the highest peak for delta *K* = 2 in the plots of L (K) vs. delta, confirming a likely allocation of the cauliflower pyramided lines into two subgroups. The individual membership coefficient at *K* = 2 had maximum mean probability of likelihood value of L (*K*) = −158.84, and the result of the structure analysis classified the lines in two groups. Individuals 1 and 2 were clustered in a group, while individuals 3, 4, and 5 were clustered in the other group (Figure 6). There were no admixed individuals.

### Agro-Morphological and Quality Traits of Pyramided Lines

**Figure 7:**
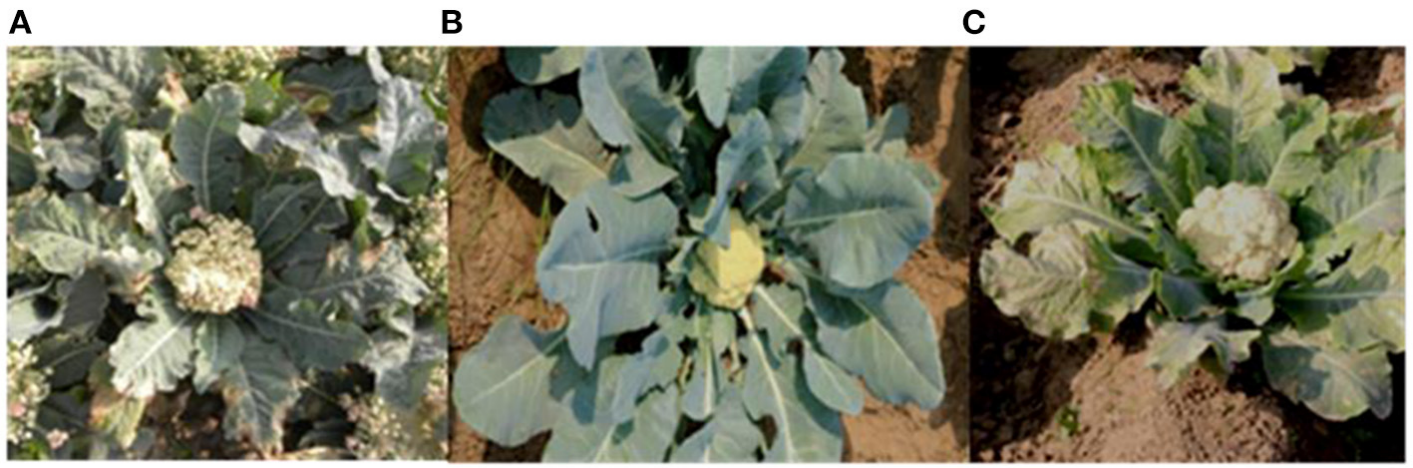
Morphological view of Pusa Meghna (susceptible) and improved Pusa Meghana (resistant to both black rot and downy mildew at curd maturity stage during evaluation of BC_2_F_2:3_ lines; **(A)** black rot-susceptible Pusa Meghna **(B)** improved Pusa Meghna (BC_2_F_2:3-7-16_) **(C)** downy mildew-susceptible Pusa Meghna.

After phenotypic screening of the pyramided lines for black rot and downy mildew resistance, five BC_3_F_2:3_ lines were evaluated for quality and yield-related parameters for 2 years. The morphological view of susceptible Pusa Meghna and improved Pusa Meghna is shown in Figure 7. The observations were analyzed in RBD, and mean values of 5 lines are presented in Table 7. The pyramided lines did not differ significantly from recurrent parent Pusa Meghna for any of the traits. In both years of evaluation, the pyramided lines BC_2_F_2:3-7-16_ and BC_2_F_2:3-7-33_ exhibited high yield at par with Pusa Meghna (172.54/176.36 q/ha) with marginal differences. Also, these lines did not differ significantly from Pusa Meghna with respect to curd weight, curd yield, and vitamin C content. The line BC_2_F_2:3-7-33_ exhibited higher yield (182.5/182.78 q/ha) compared with Pusa Meghna (172.54 q/ha) in both years. This line also exhibited higher vitamin C content (48.56/47.67 mg/100 g FW) compared with that of recurrent parent Pusa Meghna (45.76/45.34 mg/100 g FW) in both years.

**Table 7:** Mean values of morphological, yield, and quality traits of two resistant genes pyramided BC_2_F_2:3_ lines along with parents.

**Parents/Pyramided lines**	**Plant height (cm)**		**Days to 50%** ** curd maturity**		**Curd angle (** ^****°****^ **)**		**Curd weight (g)**		**Curd yield (q/ha)**		**Vitamin C** ** (mg/100 g FW)**	
	**Year I**	**Year II**	**Year I**	**Year II**	**Year I**	**Year II**	**Year I**	**Year II**	**Year I**	**Year II**	**Year I**	**Year II**
Pusa Meghna	42.63	41.77	105.69	103.65	25.69	26.06	520.36	532.02	172.54	176.36	45.76	45.34
BR-161	50.36	49.34	110.36	112.62	36.45	37.12	214.36	215.16	71.56	71.32	26.58	27.89
BR-2	40.36	40.74	95.68	96.37	39.45	38.87	206.58	210.43	69.87	69.75	30.14	30.74
BC_2_F_2:3-7-9_	44.63	42.86	104.69	106.39	27.56	27.23	545.95	516.46	180.84	171.20	42.69	45.00
BC_2_F_2:3-7-16_	41.36	42.21	103.25	101.80	29.45	28.03	514.26	531.79	170.55	176.28	46.25	43.28
BC_2_F_2:3-7-33_	40.69	40.21	100.25	98.30	28.74	29.29	550.69	551.36	182.5	182.78	48.56	47.67
BC_2_F_2:3-7-52_	39.45	38.47	95.87	96.81	30.25	30.70	500.36	506.1	165.9	167.76	40.36	39.86
BC_2_F_2:3-7-72_	40.26	38.19	100.36	99.70	28.74	28.74	522.36	517.21	170.2	171.46	44.69	43.72
CD at 0.05	4.53	4.86	5.56	4.31	2.74	2.58	29.73	16.57	18.95	5.48	3.71	5.42
CV (%)	6.09	6.58	3.11	2.39	5.09	4.74	3.79	2.09	7.31	2.09	5.21	7.58

## Discussion

Cauliflower production is hindered by many biotic stresses, among which black rot and downy mildew impose huge yield and quality losses (Singh et al., 2011; Saha et al., 2014, 2020; Verma and Singh, 2018). These challenging issues could be overcome by cultivating resistant variety(s) (Farinhó et al., 2004, 2007; Carlier et al., 2012; Saha et al., 2020). In conventional breeding, introgression of resistant gene(s) involves patho-phenotypic selection. It is a challenging and time- and resource-consuming process. The success of conventional breeding also depends on the accuracy of disease scoring, favorable environmental conditions for disease development, and availability of appropriate virulent pathogenic strain (Sundaram et al., 2014).

Marker-assisted selection is a quick, simple, and effective method for the improvement of susceptible genotypes (Lee J. et al., 2015; Lee Y. G. et al., 2015). However, recurrent parent selection in the backcross breeding programme is vital for marker-assisted breeding (Ye and Smith, 2010). In marker-assisted selection, resistant gene-linked markers help in introgression of the identified gene(s) into recipient cultivars. DNA marker technology can tag novel resistant gene(s) efficiently and provide a straight forward path for identifying and transferring gene(s) into various crops (Gu et al., 2013; Patroti et al., 2019). In previous studies, MAS has been performed to few vegetable crops such as tomato for resistance to tomato leaf curl virus (ToLCV) (Kumar et al., 2014; Prasanna et al., 2014), late blight, and root knot nematode (Kumar et al., 2019); and in pepper for resistance to PVY, TSWV, and PMMoV (Özkaynak et al., 2014). Many QTLs/gene(s) have been reported for both black rot and downy mildew diseases in cauliflower; however, there has been no report of transferring these genes(s)/QTLs through MAS. Therefore, the similar strategy was followed in this study to transfer black rot- and downy mildew-resistant genes in susceptible commercial variety Pusa Meghna along with phenotypic selection for agro-morphological traits.

In the donor parent BR-161, the *Xca1bo* gene showed complete dominance against *Xcc* race 1 and in parent BR-2, the *Ppa3* gene also showed dominance against the downy mildew disease (Singh et al., 2012; Saha et al., 2014). SCAR marker ScOPO-04_833_ and SSR marker BoGMS0624 were used to identify double positive (*Xca1bo* and *Ppa3*) plants in pyramided lines. The highly polymorphic nature of both markers made it a very effective tool to transfer *Xca1bo* and *Ppa3* resistant genes into susceptible cauliflower variety Pusa Meghna. In the BC_2_F_2_ population, both the linked markers segregated in expected Mendelian ratio, which was similar to the results of Tanweer et al. (2015), Miah et al. (2017), and Chukwu et al. (2020). However, the strong linkage of markers with the target gene is the main principle for conducting a successful MAS study (Tanweer et al., 2015).

In this study, polymorphic SSR markers were used for background selection to recover recurrent parent genome in a minimum number of backcross generations. Hence, a set of microsatellite markers that showed polymorphism between the donor and recurrent parents was used in the selection process of the recurrent parent (Pusa Meghna) genome at each backcross generation. The contribution of the recurrent parent genome at each generation of backcrossing was also estimated. In the improved lines, major part of the recurrent parent DNA segment was recovered, but in some of the improved lines, some chromosomal portions could not be recovered. Few heterozygous DNA segments were also found in some of the improved lines. The results are in line with those of Tian et al. (2006) and Hirabayashi et al. (2010). This study was helpful for getting high recovery of the recurrent parent genome and introgression of target genes into Pusa Meghna following two backcrosses and one selfing. Tanweer et al. (2015) described that full recovery of the recurrent parent genome segment is possible only in the BC_3_F_2_ generation and not in the BC_2_F_2_ generation. During transfer of multiple genes, at least three backcrosses are needed to recover the recurrent parent genome (Shu, 2009).

All the BC_2_F_2:3_ lines derived from the BC_2_F_2_ population showed strong resistance to both black rot (*Xcc* race 1) and downy mildew diseases. In India, in the case of the black rot disease, three races (races 1, 4, and 6) have been indentified of which races 1 and 4 are predominant (Singh et al., 2016). In this study, only *Xcc* race 1 was used, and the pyramided lines showed race-specific resistance. It is important to test the pyramided lines against other isolates/races to assess the spectrum of resistance. There is no report of pathogenic variability for downy mildew causing pathogen *H. parasitica* in India, and the pyramided lines showed resistance to *H. parasitica* Delhi isolate. Moreover, the yield level of these lines also did not vary significantly from that of the parent Pusa Meghna, indicating no yield penalty associated with presence of resistant genes. The pyramided lines BC_2_F_2:3-7-16_ and BC_2_F_2:3-7-33_ were found to be superior based on their high level of resistance to both black rot and downy mildew diseases, high yield, and vitamin C content. Therefore, these improved resistant pyramided lines would offer yield improvement and provide great advantage in areas where these two diseases are highly prevalent. The introgression of resistant genes for both black rot and downy mildew diseases and higher recovery of the genome of the recurrent parent in improved pyramided lines (BC_2_F_2:3-7-33_ and BC_2_F_2:3-7-16_), and higher yield are the significant achievements of this research, which will go a long way in reducing the use of chemicals, thus protecting the environment from being polluted and reducing pesticide residues from the produce, making it safe for human consumption. Besides, using the target-resistant genes in the foreground selection, the cost as well as the time for recovering the desirable recombinants can also be minimized to a great extent (Tanweer et al., 2015).

A higher recovery of desirable improved plants of the pyramided lines BC_2_F_2:3-7-33_ and BC_2_F_2:3-7-16_ was obtained because of phenotypic-based selection in each backcross generation. The current strategies of phenotypic and marker-based selection are consistent with the findings of Joseph et al. (2004), Gopalakrishnan et al. (2008), Tanweer et al. (2015), and Patroti et al. (2019), who adopted the phenotypic and marker-assisted selection for the target traits. In this study, the phenotypic-based selection process was adopted from the BC_1_F_1_ generation onward for the agro-morphological traits while screening a large number of backcross plants. Improved resistant pyramided lines exhibited an almost identical agro-morphological performance in the field, which was at par with the recurrent parent Pusa Meghna, and even the microstructure analysis classified these lines into two groups. There were no positive or negative interactions observed between both the resistant genes as observed in previous study of Sundaram et al. (2008); Hari et al. (2011), and Balachiranjeevi et al. (2015). The present results proved the efficiency of the phenotypic selection practice. Moreover, BC_2_F_2:3-7-16_ (*Xca1boXca1boPpa3Ppa3*) and BC_2_F_2:3-7-33_ (*Xca1boXca1boPpa3Ppa3*) displayed very compact, white, good quality curds similar to those of the recurrent parent Pusa Meghna. It is, therefore, evident from this study that marker-assisted breeding can be successfully performed for introgressing new genes into popular cauliflower variety. The improved pyramided lines developed in this study can be further evaluated in multi-location trials under All India Coordinated Research Project on Vegetable Crops (AICRP) for release as an improved variety to be grown by Indian farmers for increasing their income and providing chemical free fresh cauliflower curds to consumers. Further, these pyramided lines can be used in their own right as a potential variety(s) and/or donor to breed multiple disease-resistant cauliflower variety(s) and hybrids.

## Data Availability Statement

The datasets generated for this study can be found in online repositories. The names of the repository/repositories and accession number(s) can be found in the article.

## Author Contributions

PS and PK designed the experiments. PS, CG, NDS, and MS performed the experiments. PS and CG developed and phenotyped the population. CG genotyped the population. CG, NDS, and AV analyzed the data. PS and AV wrote the manuscript. PK edited and improved the manuscript. BST assisted in field facilities and edited the manuscript. All authors contributed to the article and approved the submitted version.

## Conflict of Interest

The authors declare that the research was conducted in the absence of any commercial or financial relationships that could be construed as a potential conflict of interest.

## Publisher's Note

All claims expressed in this article are solely those of the authors and do not necessarily represent those of their affiliated organizations, or those of the publisher, the editors and the reviewers. Any product that may be evaluated in this article, or claim that may be made by its manufacturer, is not guaranteed or endorsed by the publisher.
