# Supplementary material for: Marker-Assisted Pyramiding of Downy Mildew-Resistant Gene Ppa3 and Black Rot-Resistant Gene Xca1bo in Popular Early Cauliflower Variety Pusa Meghna
Source: Front Plant Sci. 2021 Aug 23;12:603600. doi: 10.3389/fpls.2021.603600 (PMC8420869; doi:10.3389/fpls.2021.603600)
Supplement: Supplementary file 1 [file Table_1.DOCX]

| No. | Primer | Forward Sequence | Reverse Sequence | Chromosome | Pusa Meghna vs. BR161 | Pusa Meghna vs. BR-2 | Amplicon size in Pusa Meghna | Ampicon size in BR-161 | Amplicon size in BR-2 |
| --- | --- | --- | --- | --- | --- | --- | --- | --- | --- |
| 1 | BoGMS1164 | _CGATTCAAACTCAAACCAAC_ | _AATAAAGAGACAGGGCGG_ | 1 | M | P | 200 | 200 | 220 |
| 2 | BoGMS0514 | _CTTCTTCCACGCTAAACATC_ | _GGTCTATTCTTTGATTCGGTT_ | 1 | P | M | 251 | 235 | 251 |
| 3 | BoGMS1322 | _CTCTCCAATCCTTCTTCTCAC_ | _CCACCTTCTCCACTAATAACC_ | 1 | M | P | 124 | 124 | 116 |
| 4 | BoGMS1042 | _ATAGTGAATAATGGAAGGCTG_ | _GAGAGAGGAGAGAACAGAGGA_ | 1 | M | P | 183 | 183 | 172 |
| 5 | BoGMS0661 | _ATTTGGATAGTGGATGGTG_ | _GAAGACATTAGGGATTGTGAA_ | 1 | P | P | 150 | 135 | - |
| 6 | BoGMS1162 | _AAATCTGACAAAGGCGAAA_ | _AGAGAGAAAGGAGAGCCAA_ | 1 | P | P | 136 | 136 | - |
| 7 | BoGMS1515 | _GTGAGAGAAGCAAACCCTT_ | _ACCAGCAATAGAGATTCAACA_ | 2 | M | P | 330 | 330 | 320 |
| 8 | BoGMS0447 | _ATTCCACGATTCTCTATGTTT_ | _GATTCATTCTGGTCGGGT_ | 2 | M | P | 175 | 175 | - |
| 9 | BoGMS0985 | _CAGTTCTCTCATTGGTATGCT_ | _CATCTTGTCATCTGCCTTG_ | 2 | P | P | 130/140 | 125/140 | 120 |
| 10 | BoGMS0726 | _GTTCCGAGGGTTGTTCTT_ | _CCATCAGGTTCAGCCATAC_ | 2 | M | P | 215 | 215 | 225 |
| 11 | BoGMS0164 | _AGAGACACACACACACACACA_ | _AAACATAAACAAACCGAAAGG_ | 2 | M | P | 347 | 347 | 335 |
| 12 | BoGMS0665 | _TCAGAGATGAACAAGAAGCAC_ | _GCGAACCTTTCCCAACCT_ | 2 | M | P | 132 | 132 | 117 |
| 13 | BoGMS0493 | _GTTACCTCCGAAATACACCTC_ | _GCCACTTCATCTCTATCACTC_ | 2 | P | M | - | 230 | - |
| 14 | BoGMS1596 | _GCACCAAGTAAAGTAAGGGAG_ | _GTAAGTAAATGAAGCAAGCGA_ | 2 | P | M | 195 | 195/207 | 195 |
| 15 | BoGMS0717 | _GAGAAACCTATCCTGCTCAC_ | _CTTATCAACGCACAACGAC_ | 3 | M | P | 210 | 210 | 233 |
| 16 | BoGMS0702 | _CGTAATGGTGAAGATACTCGG_ | _TCTAATCAAGAGCGTGTGGT_ | 3 | M | P | 285 | 285 | 295 |
| 17 | BoGMS0767 | _AAACAAGTCAGATTCACCAAA_ | _CTCTTCACCACTACCACAGTC_ | 3 | M | P | 100 | 100 | 114 |
| 18 | BoGMS1360 | _GAGACCAGAGAAGGAGGAAC_ | _CACTCACTATCACACACACTCA_ | 3 | M | P | - | - | 318 |
| 19 | BoGMS0348 | _CGTGAGTGCTTCCTCTGT_ | _TCCTTTGTCATCTTCTCCAA_ | 3 | P | M | 251 | - | 251 |
| 20 | BoGMS0407 | _ATGGTCGCTGCCTTACCC_ | _GCACAATAATACAACTGAAACT_ | 3 | P | P | 366 | 346 | 346 |
| 21 | BoGMS1464 | _CTGATGAACGGAGACACAG_ | _AAGCAAAGCAGAGCATAAAC_ | 3 | M | P | 274 | 274 | 254 |
| 22 | BoGMS1235 | _ATTCATCATCTCTCTCGGAA_ | _TAAGGCTCATCTCAACTCTCA_ | 3 | P | M | 173 | 150 | 173 |
| 23 | BoGMS0374 | _CATCTTCATCTCACACAAACA_ | _ATAACCGAAATAGCAAAGTAG_ | 3 | P | M | 373 | - | 373 |
| 24 | BoGMS0501 | _ATGATGAGTTTGCTCGTTAGG_ | _AAATCCTTCCTCCTTTCAC_ | 4 | M | P | 254 | 254 | 244 |
| 25 | BoGMS0282 | _CCCTTGTAGAGAGAGAGAGGA_ | _AAACGAAATAAGATGACGAGA_ | 4 | P | M | 230 | 210 | 230 |
| 26 | BoGMS0573 | _TTTGAGGTATTGTAGCAGATT_ | _AGCATTTGTAGTTGAGGACAG_ | 4 | P | P | - | 347 | 347 |
| 27 | BoGMS0836 | _CATAAACACACCGAACAAGAC_ | _ACGCAATGACACACATACAC_ | 4 | P | P | 136 | 146 | 146 |
| 28 | BoGMS1224 | _TCTGAGCCATTGATTGATTT_ | _CGAGGAAGAGAAGAGAAGAGA_ | 4 | M | P | 282 | 282 | 272 |
| 29 | BoGMS1049 | _CCACGGTCACTTCTCTATTT_ | _CTCTGAACCAACTCCATCTCT_ | 4 | P | P | 345 | 360 | 360 |
| 30 | BoGMS0632 | _ATCATCGTCCTCTTCTTCTTC_ | _TATCATCCTTATTGGGTCTC_ | 6 | P | M | 190 | 190/200 | 190 |
| 31 | BoGMS0742 | _TCTCTCTCCTCTTTGCTTTG_ | _GTATCAGACATTATTCACACGA_ | 6 | M | P | 220 | 220 | 240 |
| 32 | BoGMS1186 | _GACTGGAACGACAACGACT_ | _GCGGAGGTAGATTAGGGA_ | 6 | M | P | 174/190 | 174/190 | 160 |
| 33 | BoGMS1020 | _CACACCCGCAGTCTCTAC_ | _CCATTCTTCTTCTTCTTCCC_ | 7 | P | M | 358 | 377 | 358 |
| 34 | BoGMS0929 | _TCAGACCCAAAGCCAGTT_ | _TTGTGGAAGATGAAACCATT_ | 7 | P | M | 220/244 | 220 | 220/244 |
| 35 | BoGMS1486 | _AAATGTGTTCTTGGTGATG_ | _AGGAGGGTAAGTTGGTGATT_ | 7 | P | P | 220 | - | 212 |
| 36 | BoGMS0582 | _CCTGAGTCTTGGAGCCTT_ | _TCGTTATTAGATTTGAGTATTTG_ | 7 | P | M | 307 | 315 | 307 |
| 37 | BoGMS1495 | _TAACACTGAAACACATTGGCT_ | _GTGAGAAAGATGACGAAGATG_ | 7 | M | P | 382 | 382 | 368 |
| 38 | BoGMS0545 | _CCTCTGTTTCTTTGCTCTTTG_ | _GATTCATTGTGTGTGTGATGT_ | 7 | P | P | 245/260 | 245 | 245 |
| 39 | BoGMS0168 | _GTCTTGTATGAAGCCCAGTAG_ | _AGAGGAAGTGTCGGGAAG_ | 7 | P | P | 265/305/310 | 295 | 295 |
| 40 | BoGMS0627 | _TGCCACCTTATTTGGAGA_ | _GAGACTACAGGGAGGAAGAAA_ | 7 | P | P | 305 | 295 | 295 |
| 41 | BoGMS0537 | _AAGACAACATCCCTGAAGAAC_ | _GAGAGTCGCTGAACTAAAGAA_ | 7 | M | P | 230 | 230 | 210 |
| 42 | BoGMS0927 | _ACCAGAGAAGGCATACATAGA_ | _CGAAGGAGTTTGTGAGGATAA_ | 8 | P | P | 172 | 160 | 160 |
| 43 | BoGMS0364 | _CCTGTCTTTGCTCATCTATTT_ | _GCGTTAGGTTGTTGTAGGATT_ | 8 | M | P | 132 | 132 | - |
| 44 | BoGMS1145 | _CTTCTCTCTTCGCATCATAAC_ | _GTCCCTCTCCTCTCTCTCTCT_ | 8 | M | P | 345 | 345 | - |
| 45 | BoGMS0812 | _GCTGGCACATAGTTGTAATG_ | _CTCATCTCCTCTGCTGGA_ | 8 | M | P | 230 | 230 | 215 |
| 46 | BoGMS0808 | _GTCTCCTCCACCATTATCTTT_ | _GACCTCGTGTTTCCTTGA_ | 8 | P | P | 145 | 130 | 120 |
| 47 | BoGMS0674 | _ATTCTGGTAGTTGATTTGGG_ | _TCTATTCACAGGCTAACGG_ | 9 | P | P | 332 | 345 | 345 |

**Supplemental Table 1**: List of polymorphic SSR markers used in the present study
